# Effect of Graphene oxide or Functionalized Graphene Oxide on the Copolymerization Kinetics of Styrene/n-butyl Methacrylate

**DOI:** 10.3390/polym11060999

**Published:** 2019-06-04

**Authors:** Ioannis S. Tsagkalias, Afrodite Vlachou, George D. Verros, Dimitris S. Achilias

**Affiliations:** Laboratory of Polymer and Dyes Chemistry and Technology, Department of Chemistry, Aristotle University of Thessaloniki, GR-541 24 Thessaloniki, Greece; itsagkal08@gmail.com (I.S.T.); afrodite.vlachou@gmail.com (A.V.); gdverros@gmail.com (G.D.V.)

**Keywords:** copolymerization kinetics, graphene oxide, styrene, n-butyl methacrylate, modeling

## Abstract

Nanocomposite materials based on copolymers of styrene and n-butyl methacrylate with either graphene oxide (GO) or functionalized graphene oxide (F-GO) were synthesized using the in-situ bulk radical copolymerization technique. Reaction kinetics was studied both experimentally and theoretically using a detailed kinetic model also taking into account the effect of diffusion-controlled phenomena on the reaction kinetic rate constants. It was found that the presence of GO results in lower polymerization rates accompanied by the synthesis of copolymers having higher average molecular weights. In contrast, the presence of F-GO did not seem to significantly alter the conversion vs time curves, whereas it results in slightly lower average molecular weights. The first observation was attributed to side reactions of the initiator primary radicals with the hydroxyl groups on the surface of GO, resulting in lower initiator efficiency, whereas the second to grafted structures formed from copolymer macromolecules on the F-GO surface. The copolymerization model predictions including MWD data were found to be in satisfactory agreement with the experimental data. At least four adjustable parameters were employed and their best-fit values were provided.

## 1. Introduction

Graphene is believed to be the “thinnest and strongest material known so far” and has recently attained significant research interest due to its exceptional mechanical, electrical, thermal and optical properties. It has a Young’s modulus near 1 TPa. These properties make graphene one of the most popular nano-additives for the development of functional and structural graphene-reinforced polymer nanocomposites [1].

Graphene can be obtained from the exfoliation of graphite sheets. However, it is easier to get graphene oxide (GO) sheets from the exfoliation of graphite oxide. The latter can be produced from the oxidation of graphite using concentrated acids in the presence of strong oxidants and consists of many oxygen-containing groups, such as carboxyl, hydroxyl and epoxy groups in the basal planes and edges. Moreover, functionalized graphene oxide (F-GO) could be produced by reacting the surface hydroxyl groups of GO with a silane which has methacrylate groups [2,3,4,5,6].

A feasible way to incorporate graphene, GO or F-GO in a nanocomposite polymer matrix is in-situ polymerization as it ensures good dispersion of the nano-additive in the polymer matrix and improved final product properties.

This technique has been applied to produce non-covalent graphene-based nanocomposites of several polymers such as poly(methyl methacrylate) PMMA [7,8,9,10,11,12,13]. Moreover, nanocomposites with enhanced thermal properties were also obtained when using alkyl functionalized GO [11].

Although synthesis by in situ polymerization and the study of properties of homopolymers based on nanocomposites have been extensively studied in the literature [14,15,16,17,18,19,20,21,22,23,24,25], the publications on copolymers, such as the butyl methacrylate-styrene GO or F-GO nanocomposites, are rather limited.

In particular, the butyl methacrylate-styrene copolymers are the main components of many solvent-based automotive coating formulations. They are extensively used as hindered resins providing excellent protection against chemical and mechanical attack [26]. Due to their ultimate properties, the production of butyl methacrylate-styrene copolymers has been the subject of extensive investigation in the past [26,27,28,29,30]. Its similar monomer reactivity ratios, determined from composition experiments as r_ST_ = 0.61 and r_BMA_ = 0.42 [26], guarantee the synthesis of a statistical copolymer (if both reactivity ratios were equal to 1 then an ideal random copolymer could be formed). In a previous publication by our group [31], the effect of adding organo-modified clays on the polymerization kinetics and properties of P(S-*co*-BMA) copolymers was investigated.

This work is a continuation of our previous studies on the effect of adding either GO or F-GO on the polymerization kinetics of styrene or n-butyl methacrylate [21,25,32]. The aim of this research was to investigate both theoretically and experimentally the in-situ bulk free radical copolymerization of n-butyl methacrylate with styrene in the presence of several amounts of either graphene oxide or functionalized graphene oxide. It should be pointed out here that this is the first time that the effect of GO or F-GO on the copolymerization kinetics is presented in the literature. Two copolymer compositions were investigated, one rich in styrene and the other in butyl methacrylate. Polymerization kinetics was studied gravimetrically which had the advantage of providing absolute measurements of conversion versus time, while samples at different time intervals are available. The average molecular weights and the molecular weight distribution of the materials formed were measured with Gel Permeation Chromatography (GPC).

## 2. Materials and Methods

### 2.1. Materials

The monomers used were styrene (S) and n-butyl methacrylate (n-BMA), both purchased from Alfa Aesar (Haverhill, MA, USA) with a purity of ≥ 99%. The inhibitor was removed by passing it, at least twice, through a disposable inhibitor-remover packed column (Aldrich, Hamburg, Germany). Benzoyl peroxide (BPO) was used as a free radical initiator (purity > 97%), which was provided by Alfa Aesar and purified by fractional recrystallization twice from methanol (CHEM-LAB, Zedelgem, Belgium). Dichloromethane and methanol used in the dissolution and reprecipitation of the polymer were purchased from CHEM-LAB. Graphite powder was purchased from Sigma-Aldrich. All other chemicals used were of analytical grade and were used as received without further purification.

### 2.2. Synthesis of Graphite Oxide and Functionalized Graphite Oxide

Graphite Oxide was prepared from the oxidation of graphite powder according to the Hummers method. Details can be found elsewhere [21,25,32].

For the preparation of the functionalized graphite oxide, a silane-modifying agent (3-methacryloxypropyltrimethoxysilane-MPS) was used, and the whole procedure can be found in detail in the literature [25,32].

### 2.3. Synthesis of P(S-co-BMA)/GO and P(S-co-BMA)/F-GO Nanocomposites by the In-Situ Bulk Radical Polymerization Technique

Two copolymers with different initial monomer ratios were prepared and studied here. One was rich in butyl methacrylate, while the other in styrene. These were given the code names P(S-*co*-BMA) 20:80 and P(S-*co*-BMA) 60:40 denoting a 20:80 or 60:40 molar ratio of S:BMA, respectively. These specific monomer ratios were selected based on a previous publication by our group [31]. Once the proper monomer mixture (i.e., S with BMA) was prepared, graphite oxide or functionalized graphite oxide was added and the suspension was positioned to ultrasonication (Transsonic 460H ultrasonic bath from Elma, Singen, Germany) for 1 h, so as to have a satisfactory colloidal dispersion of GO to the solution, while exfoliation of graphite oxide to graphene oxide started. In the final mixture, the initiator BPO (concentration 0.03 M) was added. The mixture was degassed by passing nitrogen and was then immediately used. Three different percentages of GO or F-GO relative to the monomer mixture were employed, namely 0.1, 0.5 and 1.0 wt%. Neat copolymers were also synthesized under the same experimental conditions and used as reference materials.

The bulk, free radical copolymerization was carried out in small specially-designed test-tubes by heating the initial monomer–GO–initiator mixture at 80 °C for a suitable time. Special care was taken to keep the reaction temperature constant during copolymerization using small co-monomer amounts. The reaction temperature and initial initiator concentration were kept the same in all experiments. Accordingly, 1 mL of the pre-weighed mixture of monomers with the initiator and each amount of GO were placed into a series of 10 small test-tubes. After degassing with nitrogen, they were sealed and placed into a pre-heated bath. Each test tube was removed from the bath at the pre-specified time intervals and was immediately frozen after the addition of a few drops of hydroquinone, in order to stop the reaction. The product was isolated after dissolution in CH_2_Cl_2_ and re-precipitation in MeOH. Subsequently, all isolated materials were dried to a constant weight in a vacuum oven at room temperature. All final samples were weighed and the degree of conversion was estimated gravimetrically. Details of the experimental technique can be found in the literature [25,32]. All experiments were repeated at least twice and the variation of the data was not more than 2%.

### 2.4. Measurements

The final molecular weight distribution (MWD) and the average molecular weights of pristine copolymers and their nanocomposites with either GO or F-GO were determined by *Gel Permeation Chromatography (GPC)*. The instrument used was the PL-GPC 50 Plus, from Polymer Laboratories, and included an isocratic pump, a differential refractive index detector, and three PLgel 5 μ MIXED-C columns in series. Samples were dissolved in tetrahydrofuran (THF) at a constant concentration of 1 mg mL^−1^. After filtration, 200 μL of each sample was injected into the chromatograph. The elution solvent was THF at a flow rate of 1 mL min^−1^, and the entire system was kept at a constant temperature of 30 °C. Calibration of GPC was carried out with standard poly(methyl methacrylate) samples (Polymer Laboratories) with peak molecular weight ranging from 690 to 1,944,000 g mol^−1^ and the universal calibration method using appropriate Mark-Houwink constants.

^1^H NMR spectra of copolymers were obtained with a Bruker spectrometer operating at a frequency of 300 MHz for protons. A mixture of deuterated trifluoroacetic acid (DTFA) and chloroform in a ratio 3/1 *w*/*w* (DTFA/CDCl_3_) was used as a solvent in order to prepare solutions of 5% *w*/*v*. The number of scans was 10, and the sweep width was 6 kHz.

## 3. Theoretical Section

A fairly general kinetic mechanism to account for butyl methacrylate-styrene free radical copolymerization in the presence of GO or F-GO includes the following elementary reactions [33]:

Chemical initiation: $I\overset{k_{d}}{\to }2R^{•}$
Thermal initiation of styrene: ${3M}_{2}\overset{k_{\mathrm{th}}}{\to }{3R}_{0,1}$
Chain Initiation: $R^{•}+M_{j}\overset{k_{\mathrm{Ij}}}{\to }R_{2-j,j-1}^{j};j=1,2$
Propagation: $R_{p,q}^{i}+M_{j}\overset{k_{pij}}{\to }R_{p+2-j,q+j-1}^{j};i,j=1,2$
Chain transfer to monomer: $R_{p,q}^{i}+M_{j}\overset{k_{fmij}}{\to }R_{2-j,j-1}^{j}+D_{p,q};i,j=1,2$
Chain transfer to polymer: $R_{p,q}^{i}+D_{x,y}\overset{k_{fpij}}{\to }R_{x,y}^{i}+D_{p,q};i,j=1,2$
Termination by combination or disproportionation: $R_{p,q}^{i}+R_{x,y}^{j}\left\{\begin{array}{c} \overset{k_{tcij}}{\to }D_{p+x,q+y}\\ \overset{k_{tdij}}{\to }D_{p,q}+D_{x,y} \end{array}\right.;i,j=1,2$

Symbol D stands for “dead” polymer, and symbol R stands for radicals. The two subscripts (*p,q*) denote a copolymer chain containing p units of monomer 1 (BMA) and q units of monomer 2 (styrene). The superscripts (*i.j*) refer to the ultimate monomer unit in the radical chain. It should be noted that the ultimate unit in the radical chain could be either type 1 (BMA) or type 2 (styrene).

Following our previous work [25], chain transfer to polymer reactions were introduced to account for the co-polymerization reaction occurring at the nano-additive surface

The mass conservation of the various species G present in an isothermal batch copolymerization reactor is described by the following set of differential equations:

(1) $$\frac{d(\mathrm{VG})}{dt}=Vr_{G}$$

where the symbol G stands for the initiator (r_i_), primary radicals (r_R_), i-th type monomer (r_Mi_), free radicals of the i-th type, and “dead” polymer.

Based on the above copolymerization mechanism, one could directly derive the reaction rates which are given in the Supplementary Materials section together with the variation of the reaction volume with time.

To calculate the mean copolymer composition as well as the average molecular weights, one has to resort to the method of moments [34,35,36,37]. The respective rate functions for the moment equations of the joint chain length-copolymer composition distribution are also given in the Supplementary Materials section.

The Number and the Weight Average Molecular Weight as well the mean copolymer composition are directly calculated from the above leading moments [34,35,36,37]:

(2) $${\bar{M}}_{n}=\frac{{\mathrm{MW}}_{1}\mu_{10}+{\mathrm{MW}}_{2}\mu_{01}}{\mu_{0}}\;{\bar{M}}_{w}=\frac{{\mathrm{MW}}_{1}(\mu_{20}+\mu_{11})+{\mathrm{MW}}_{2}(\mu_{02}+\mu_{11})}{\mu_{10}+\mu_{01}}\;\mathrm{CC}=\frac{\mu_{10}}{\mu_{01}+\mu_{10}}$$

An important issue of the butyl methacrylate–styrene copolymerization is the existence of diffusion-controlled kinetic rate constants at high conversion. Achilias and Sideridou [38] developed a comprehensive model for free radical co-polymerization based on the Smoluchowsi equation. Following Achilias and Sideridou model [38] developments and by assuming that the diffusion limitations in the propagation rate constant (glass effect) could be neglected [25], the diffusion-controlled kinetic rate constants for copolymerization are summarized in Table 1.

All the symbols are explained in the Nomenclature Section. The glass transition temperature of the copolymer (T_gp_) was calculated from the homopolymers by using the Fox equation which was extensively validated by other workers [39,40] in the field. The kinetic rate constants in the absence of diffusion phenomena for butyl methacrylate were adopted from our previous work [25] and for styrene from Cavin et al. [41]. The thermophysical properties for both monomers or the respective homopolymers such as monomer or polymer density, as well as glass transition temperature are given in full detail elsewhere [25,26,27,28,29,30,41].

The reactivity ratios r*_i_* = k_p0*ij*_/k_p0*ii*_, *i, j* = 1,2 *i*≠*j* were adopted from Fukuda et al. [27]. Moreover, by following these workers, it was assumed that the penultimate unit effect could be incorporated in the model parameters. It should be pointed out here that according to Li et al. [26], the terminal model prediction of the composition-averaged propagation rate coefficient for this specific system deviates significantly from experimental values. Therefore, the penultimate model was used in this investigation for the estimation of the adjustable parameters. However, in the modeling equations (presented in the Supplementary Materials), we preferred to use the terminal model in order to minimize the number of parameters used. The penultimate model uses eight propagation kinetic parameters instead of only four for the terminal model.

The cross termination kinetic rate constants in the absence of diffusional phenomena (k_tc0*ij*_, k_td0*ij*_*, i, j* = 1,2 *i*≠*j*) were approximated as the geometric mean of the respective main constants for termination. Finally, the cross kinetic rate constants for transfer to monomer or transfer to polymer were directly calculated from the reactivity ratios.

A major task in this work is to calculate the entire Molecular Weight Distribution (MWD) of the produced copolymer in the presence not only of diffusion phenomena but also of transfer to polymer reactions. For this purpose, the polymerization rate functions (Equation (1)) along with the moment equations (see Supplementary Materials) and the volume equation (Equation (2)) were simultaneously solved by also taking into account the diffusion controlled reactions (see Table 1). Details are provided in our previous work [42].

## 4. Results and Discussion

### 4.1. Experimental Polymerization Kinetics

**Figure 1:**
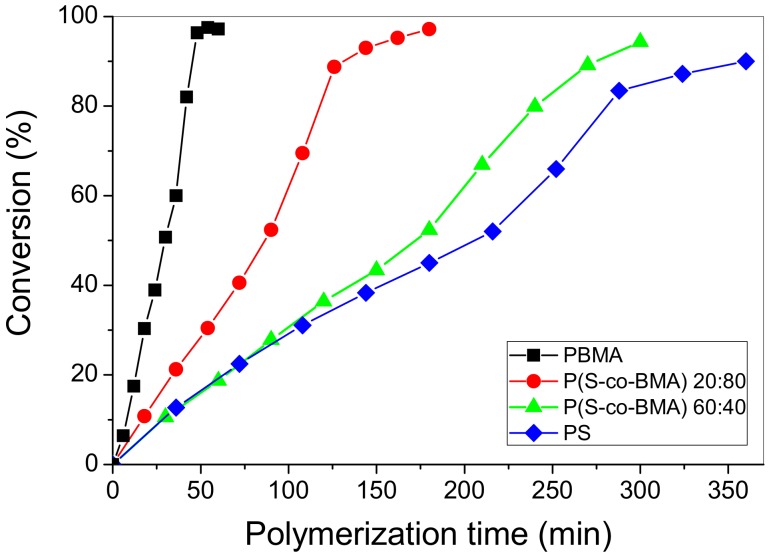
Evolution of conversion with time of the neat copolymers P(S-co-BMA) 20:80 and P(S-co-BMA) 60:40 and the homo-polymers PS [32] and PBMA [25].

The evolution of conversion with the time of the two neat copolymers (i.e., P(S-*co*-BMA) 20:80 and P(S-*co*-BMA) 60:40 appears in Figure 1. In this figure, data of the two corresponding homopolymers (i.e., PS and PBMA) is included. Conversion curves versus time follow classical radical polymerization kinetics until an almost 40% conversion, whereas afterwards an increase in the reaction rate is observed due to the well-known auto-acceleration or gel-effect. The copolymer with the higher amount of styrene presents a behavior similar to that of the PS homopolymer, whereas the copolymer reach in BMA shows a conversion curve in-between the two corresponding homo-polymers. Details on the characteristics of the conversion vs time curves can be found in the literature [25,32].

**Figure 2:**
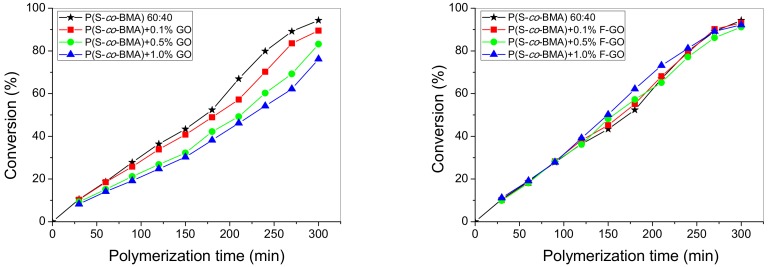
Variation of monomer conversion with polymerization time during bulk in-situ radical polymerization of styrene with n-butyl methacrylate, 60:40 at 80 °C at various graphene oxide (**a**) or functionalized graphene oxide (**b**) contents.

**Figure 3:**
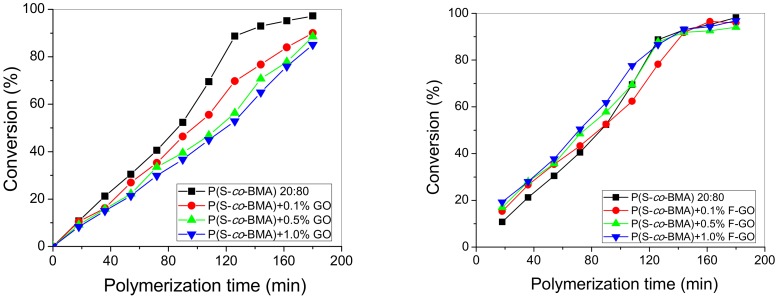
Variation of monomer conversion with polymerization time during bulk in-situ radical polymerization of styrene with n-butyl methacrylate, 20:80 at 80 °C at various graphene oxide (**a**) or functionalized graphene oxide (**b**) contents.

Τhe effect of adding GO or F-GO on the copolymerization kinetics of P(S-*co*-BMA) 60:40 and P(S-*co*-BMA) 20:80 is illustrated in Figure 2 and Figure 3, respectively.

From Figure 2a and Figure 3a, it is clear that as the amount of GO increases, the polymerization rate is reduced, resulting in lower conversion values at specific reaction times. In contrast, the addition of F-GO did not seem to significantly alter the conversion vs time curves. A similar behaviour has also been observed in the homo-polymers PS [32], PMMA [43] and PBMA [25]. The reduced polymerization rate with the amount of GO added has been attributed to the reaction of primary radicals formed from the fragmentation of the initiator with the surface functional groups of GO and mainly the hydroxyl groups, -OH. This results in reduced initiator efficiency, which directly affects the polymerization rate. Concerning the effect of adding F-GO to the polymerization kinetics, the formation of grafted polymers has been proposed to explain this behavior. Further details of this phenomenon are presented in the subsequent section using simulation results.

**Figure 4:**
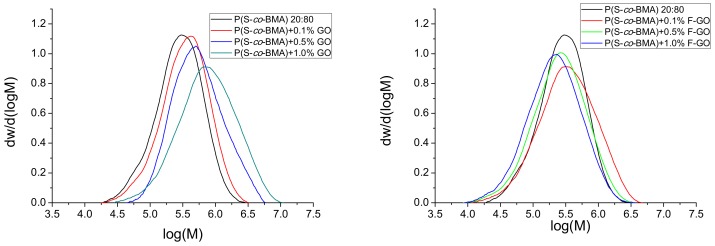
Molecular weight distribution of neat P(S-*co*-BMA) 20:80 and its nanocomposites with either GO (**a**) or F-GO (**b**) obtained after bulk in-situ radical polymerization.

**Figure 5:**
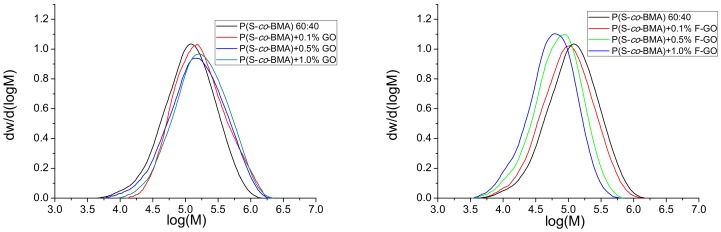
Molecular weight distribution of neat P(S-*co*-BMA) 60:40 and its nanocomposites with either GO (**a**) or F-GO (**b**) obtained after bulk in-situ radical polymerization.

Furthermore, the MWD of all copolymers and nanocomposites was measured using GPC, and results appear in Figure 4 and Figure 5 for the P(S-*co*-BMA)20:80 and P(S-*co*-BMA) 60:40, respectively.

From Figure 4a and Figure 5a, it was observed that the MWD of the copolymer was shifted to higher values as the amount of GO added increased. This was more intense in the P(S-*co*-BMA) 20:80 copolymer. Particularly, the number average molecular weight, M_n_, of P(S-*co*-BMA) 20:80 increased from 233,500 to 374,000 and 440,000 g/mol with the increased amounts of GO added. The M_n_ of the neat copolymer was measured at 193,000 g/mol. A similar increase was measured in the P(S-*co*-BMA) 60:40 copolymer with M_n_ values starting from 75,850 at 0.1 wt% GO added and increased to 91,320 and 112,850 g/mol, with 0.5 or 1.0 wt% of GO. The increase in the M_n_ of the copolymer with the amount of GO added can be explained based on the side reactions of primary radicals formed from the decomposition of the initiator, thus resulting in reduced initiator efficiency. A lower number of primary radicals formed results in a lower number of macro-radicals, which can add more monomer molecules and thus result in final copolymers with a higher chain length. More details can be found in the simulation section. The lower values measured in the copolymer with the higher amount of styrene are attributed to the lower chain-length polymers obtained after polymerization of styrene compared to those corresponding to the polymerization of PBMA [25,32].

Moreover, when using the functionalized F-GO it was found that the MWD of the copolymer was slightly affected and particularly shifted to lower values compared to neat copolymer. Particularly, the M_n_ was decreased from 196,000 to 157,000 and 132,500 g/mol for the P(S-*co*-BMA) 20:80 with the addition of 0.1, 0.5 and 1.0 wt% of F-GO. A lower corresponding decrease was measured in the P(S-*co*-BMA) 60:40, where M_n_ decreased from 73,050, to 46,820 and 38,580 g/mol with the addition of 0.1, 0.5 and 1.0 wt% of F-GO. This reduction in Mn has been attributed to the methacrylate groups incorporated into the GO surface through the functionalization process and the particular modifying agent used (i.e., MPS). Thus, it seems that some of the monomer reacts with these groups, resulting in decreased available molecules to find a macroradical and produce macromolecular chains. Moreover, the surface vinyl groups in F-GO may behave as a surface for grafting of macromolecules resulting in partially-grafted materials. This phenomenon does not directly affect the conversion vs time experimental data, since at high degrees of conversion, the tubes were broken and the material was received as it was. However, it affects the average molecular weight data, since the solubilization of the material in THF in order to perform the GPC experiments results in measuring the MWD of only the soluble part and not of that partially grafted. Indeed, some insoluble material was visual in the samples prepared for GPC measurements, when the amount of F-GO added was high.

### 4.2 Simulation Results

The behavior observed experimentally was further explained using model simulations. To account for the formation of graft structures due to polymerization of the surface double bonds introduced by the modification of the GO surface, two different strategies (model I and II) in the selection of the adjustable parameters were applied.

The adjustable parameters of the model I for neat copolymerization as well as for polymerization in the presence of GO, include the initial initiator efficiency (f_0_), a single gel effect parameter (D_p00*,ij*_ = D_p00_ in Table 1), the transfer to monomer for the butyl methacrylate kinetic rate constant (k_fm__11_), and the cage effect parameter (D_I0_ /C). In this case, the transfer to polymer kinetic rate constants was set equal to zero.

The residual termination constants A_ij_, for simplicity, were also set equal to zero. The fitted data for model I includes the monomer conversion data and the number average molecular weight at the end of polymerization.

To model the copolymerization kinetics in the presence of FGO, the transfer to the polymer kinetic rate constant was added as an adjustable parameter and the weight average molecular weight was also introduced in the fitted data (model II). Comprehensive numerical analysis methods for the non-linear regression problem were used [44].

**Figure 6:**
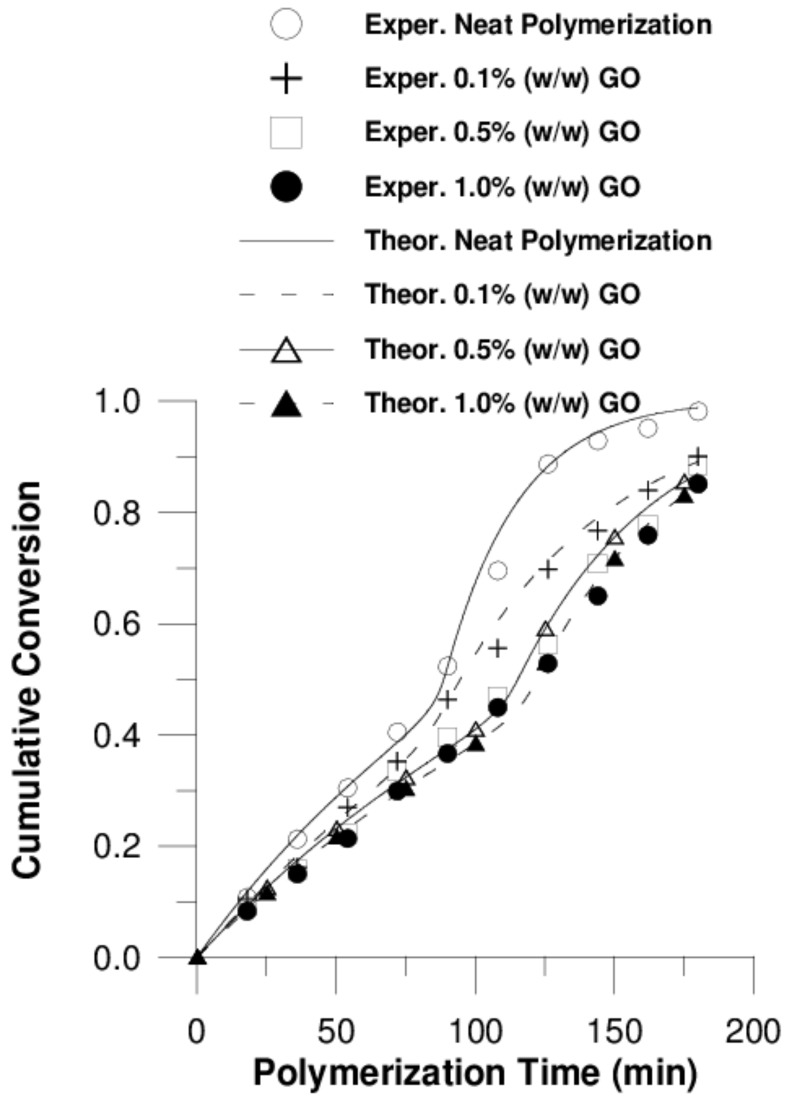
Cumulative conversion vs. polymerization time at various graphene oxide (GO) contents (Model I) for P(S-*co*-BMA) 20:80.

**Figure 7:**
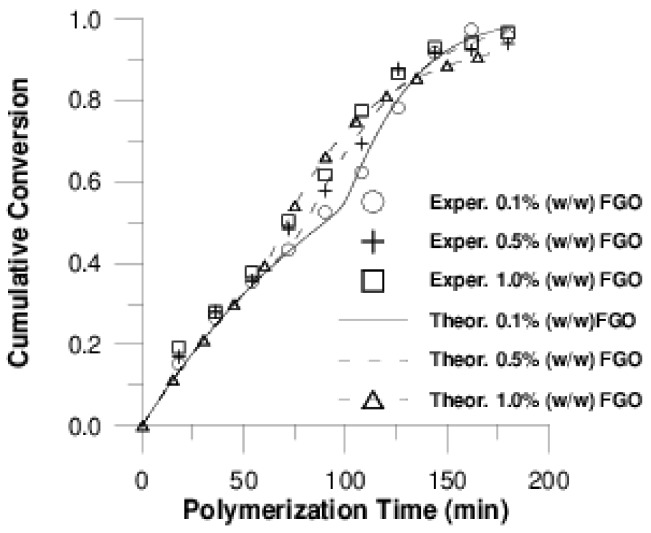
Cumulative conversion vs. polymerization time at various functionalized graphene oxide (F-GO) contents (Model II) for P(S-*co*-BMA) 20:80.

A fairly good fitting for conversion data is illustrated in Figure 6 and Figure 7. The estimated values for adjustable parameters are given in Table 2.

These values are comparable to the respective values for the neat copolymerization:

f_0_ equal to 0.8 ± 0.04, gel effect parameter (D_p00_ × 10^−17^) equal to 0.33 ± 0.03 dm^3^ mol^−1^ s^−1^, k_fm11_ was equal to 1.15 ± 0.08 dm^3^ mol^−1^ s^−1^ and the cage effect parameter (D_I0_/C) × 10^−5^ was equal to 0.33 ± 0.02.

In Table 2, the estimated values for the adjustable parameters of model II are also summarized. Moreover, as shown in Table 2, the estimated values for initiator efficiency (f_0_) remain constant and equal to unity as the F-GO concentration increases; this is a clear indication that the modified graphene oxide is not a scavenger but a polymerization reaction promoter.

There is also in Table 2, a clear tendency for the estimated value of the transfer to polymer kinetic rate constant to decrease as the concentration of F-GO increases. This behavior could be attributed to the introduced steric hindrance effects introduced by increasing the nano-additive concentration and thus leading to the enhancement of polymerization at the nano-additive surface.

These observations are in accordance with our previous work [25] for PBMA free-radical homopolymerization in the presence of either GO or F-GO.

The predicted values for number average molecular weight and for weight average molecular weight are compared with the experimental data in Table 3.

Although the experimental weight average molecular weight data (model I: neat copolymerization-addition of GO) was not used in the parameter estimation procedure, it is in satisfactory agreement with theoretical predictions (model I).

Regarding copolymer composition, the simulations for the neat copolymer result in a value equal to 25.8% mole in styrene; the respective values for polymerization in the presence of either GO or F-GO are 0.238 ± 0.003 and 0.25 ± 0.002, respectively. These values are in satisfactory agreement with the experimental ones in this work.

**Figure 8:**
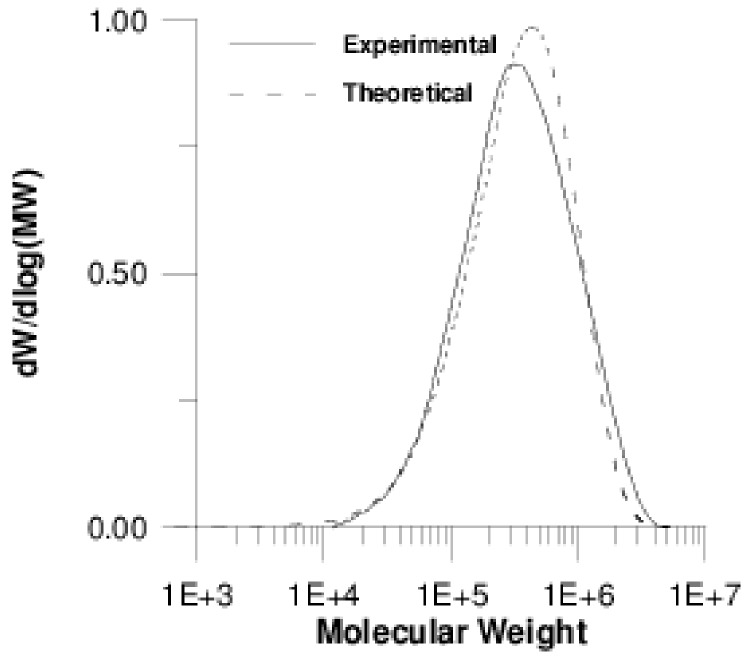
Comparison of the experimental MWD with the predicted one (Model II, 0.1 % (*w*/*w*) F-GO) for P(S-*co*-BMA) 20:80.

To further demonstrate the predictive abilities of our model, the entire MWD was calculated for the non-linear copolymerization in the presence of F-GO and compared in Figure 8 to the experimental one.

In this Figure, an excellent agreement is depicted between experimental data for the entire MWD and theoretical predictions by using model II, further validating the results of this work.

Regarding the simulation results of P(S-*co*-BMA) 60:40, an excellent fitting was directly obtained by further increasing the number of adjustable parameters. In particular, by adding as adjustable parameters the residual termination constants A*_ij_* and by increasing the number of gel effect parameters (D_p00,*ij*_), an excellent fitting to experimental data was obtained.

## 5. Conclusions

In this work, the in-situ bulk radical copolymerization of BMA with styrene in the presence of GO or functionalized graphene oxide was studied both experimentally and theoretically. From polymerization kinetics measurements, it was found that GO acts as a primary radical scavenger reducing the polymerization rate, while F-GO acts as a ‘quasi’ promoter of the polymerization reaction through the formation of grafted structures between the copolymer and the F-GO surface. Thus, it is concluded that in the case of monomers with similar reactivities, such as those studied here, the addition of GO or F-GO on polymerization kinetics is similar to that in the corresponding homo-polymers. Moreover, it seems that the addition of these nano-fillers does not affect the copolymer properties (i.e., average copolymer composition). The model predictions including MWD data and monomer conversion were found to be in satisfactory agreement with the experimental data.

## Figures and Tables

**Table 1:** Model Equations for Diffusion-Controlled Copolymerization Reactions [38].

Name	Equations
Diffusion Controlled Limitations for Termination Reactions	$k_{tij}=k_{\mathrm{te}ij}+k_{t,\mathrm{reac}ij}$
Gel Effect	$\frac{1}{k_{\mathrm{te}ij}}=\frac{1}{k_{t0ij}}+\frac{{\bar{M}}^{2}}{D_{p00ij}\exp(-b/V_{f})}$
Residual Termination	$k_{t,\mathrm{reac}ij}=A_{ij}k_{p}M$
Diffusion Controlled Limitations for Initiation Reaction (Cage Effect)	$\frac{1}{f}=\frac{1}{f_{0}}+\frac{C}{D_{I}}=\frac{1}{f_{0}}+\frac{C}{D_{I0}\exp(-{b/V}_{f})}$
Free Volume Parameters	$V_{f}=0.025+0.00048\left(T-T_{g}\right)\;\frac{1}{T_{g}}=\frac{1}{T_{\mathrm{gm}}}+X_{\mathrm{cum}}\left(\frac{1}{T_{\mathrm{gp}}}-\frac{1}{T_{\mathrm{gm}}}\right)$ $\frac{1}{T_{\mathrm{gm}}}=\sum_{i=1}^{2}\frac{w_{i0}}{T_{gmi}}$

**Table 2:** Values of Adjustable Parameters used in Simulations for P(S-*co*-BMA) 20:80 Nano-composites.

**GO (*w*/*w*)%:**	**0.1%**	**0.5%**	**1%**
f_0_	0.52 ± 0.02	0.48 ± 0.03	0.41 ± 0.02
^a^ D_p00_ × 10^−17^	0.075 ± 0.003	0.63 ± 0.03	0.63 ± 0.04
^a^ k_fm*11*_	0.97 ± 0.04	0.5 ± 0.03	0.41 ± 0.02
(D_I0_/C) × 10^−5^	5.33 × 10^−3^	0.04 ± 0.003	0.02 ± 0.002
**F-GO (*w*/*w*)%:**	**0.1%**	**0.5 %**	**1%**
f_0_	1	1	1
^a^ D_p00_ × 10^−17^	288.4 ± 10.5	1.1 ± 0.07	2.45 × 10^−3^
^a^ k_fm*11*_	0.95 ± 0.03	1.45 ± 0.11	1.81 ± 0.15
^a^ k_fp_	0.45 ± 0.03	0.41 ± 0.02	-
(D_I0_/C) × 10^−5^	31.62 ± 2.3	0.15 ± 0.01	0.014 ± 0.002

^a^ in dm^3^ mol^−1^ s^−1^.

**Table 3:** Final Product Number Average Molecular Weight (M_n_) and Weight Average Molecular Weight (M_w_) for neat P(S-*co*-BMA) 20:80 copolymer and its nanocomposites.

Sample	M_n_ × 10^−5^ (g/mol) (exp.)	M_n_ × 10^−5^ (g/mol) (Model)	M_w_ × 10^−5^ (g/mol) (exp.)	M_w_ × 10^−5^ (g/mol) (Model)
neat	1.93	1.934	5.71	4.04
0.1 wt.% GO	2.335	2.331	4.6	4.78
0.5 wt.% GO	3.74	3.740	7.73	8.026
1 wt.% GO	4.40	4.397	11.53	9.523
0.1 wt.% F-GO	1.96	1.998	5.13	5.193
0.5 wt.% F-GO	1.57	1.530	3.72	3.765
1 wt.% F-GO	1.325	1.334	3.11	2.729

## Supplementary Materials

The following are available online at https://www.mdpi.com/2073-4360/11/6/999/s1.

## Nomenclature

A_ij_	residual termination adjustable parameter between the i-th type and the j-th type radical
D_p,q,_; *D**_p,q,_*	“dead” polymer having *p* BMA units and q styrene units; its concentration
b_0_	free volume theory adjustable parameter set equal to unity
B_i_	auxiliary parameters
CC	copolymer composition
D	polydispersity
D_I0_	free volume theory pro-exponential parameter for primary radicals
$D_{p00ij}$	free volume theory pro-exponential parameter for macroradicals self-diffusion for the termination reaction between the i-th and j-th type macroradicals
C/D_I0_	cage effect parameter
f	initiator efficiency
I; *I*	initiator; its concentration
*k_d_*	initiator decomposition kinetic rate constant
*k_fmij_*	chain transfer to monomer kinetic rate constant
*k_fpij_*	chain transfer to polymer kinetic rate constant
*k_pij_*	propagation rate kinetic rate constant
*k_tij_*	overall termination kinetic rate constant
*k_t0ij_*	intrinsic termination rate constant defined at zero conversion and involving two short chains
*k_tcij_*	termination by combination kinetic rate constant
*k_tdij_*	termination by disproportionation kinetic rate constant
*k_teij_*	diffusion-controlled termination kinetic rate constant
*k_t,reacij_*	reaction-diffusion controlled kinetic rate constant
M_i_; *M_i_*	i-th type monomer; its concentration
MW	molecular weight
$\bar{M}$	radicals number average degree of polymerization
${\bar{M}}_{n}$	number average molecular weight
${\bar{M}}_{w}$	weight average molecular weight
R^•^; *R*^•^	primary radical from the fragmentation of the initiator; its concentration
R	universal gas constant
$R_{p,q}^{i}$, $R_{p,q}^{i}$	macroradical having the i-th type monomer unit, consisting of *p* BMA units and of q styrene units; its concentration
t	time
T	temperature
T_g_	glass transition temperature
V	reactor volume
w_i_	weight fraction of the i-th type monomer
$V_{f}$	free volume fraction
X_cum_	cumulative conversion
Y_i_	fractional i-th type monomer conversion
*Greek symbols*	
ε	volume contraction factor
λ_nm_	n,m moments of “live” radicals chain length distribution-chain length-copolymer composition distribution
μ_nm_	n,m moments of “dead” polymer chain length-copolymer composition distribution
ρ	density
*Subscripts*	
I	initiator
m	monomer
o	initial conditions
p	polymer
