# Supplementary material for: Effect of Graphene oxide or Functionalized Graphene Oxide on the Copolymerization Kinetics of Styrene/n-butyl Methacrylate
Source: Polymers (Basel). 2019 Jun 4;11(6):999. doi: 10.3390/polym11060999 (PMC6630914; doi:10.3390/polym11060999)
Supplement: Supplementary file 1 [file polymers-11-00999-s001.pdf]

polymers

MDPI

SUPPLEMENTARY MATERIAL

1. **Polymerization Rate Functions**

Initiator

$$\mathrm{r}_I = -k_d \mathrm{I} \qquad \text{(S1)}$$

Monomer- Fractional i-th Monomer Conversion (Y_i)

$$\mathrm{r}_{pi} = \sum_{j=1}^{2} \left(k_{pij} + k_{fmij}\right) \mathrm{M}_i \mathrm{R}^j_{0,0} + k_{th} \mathrm{M}_2^3 \delta(2-i) \qquad \text{(S2)}$$

Macromolecular Species Balance

$$\mathrm{r}_{R^\bullet} = 2\mathrm{fk_d I} - \sum_{j=1}^{2} k_{Ij} \mathrm{R}^\bullet \mathrm{M}_j = 0 \qquad \text{(S3)}$$

$$\mathrm{r}_{R^i_{n,m}} = \left(k_{iI} \mathrm{R}^\bullet \mathrm{M}_i + \sum_{j=1}^{2} k_{fmji} \mathrm{M}_i \mathrm{R}^i_{0,0} \right) \delta(n+i-2, m+1-i) + \sum_{j=1}^{2} k_{pji} \mathrm{M}_i \mathrm{R}^j_{n+i-2,m+1-i} + \sum_{j=1}^{2} k_{pji} \mathrm{M}_j \mathrm{R}^i_{n,m} - \mathrm{A}_i \mathrm{R}^i_{n,m} + \mathrm{B}_i + 3k_{th} \mathrm{M}_2^3 \delta(n+i-2, m+1-i) \qquad \text{(S4)}$$

$$\mathrm{r}_{D_{n,m}} = \sum_{i=1}^{2} \left(\mathrm{A}_i - \sum_{j=1}^{2} k_{tcij} \mathrm{R}^j_{0,0} \right) \mathrm{R}^i_{n,m} + \frac{1}{2} \sum_{i=1}^{2} \sum_{j=1}^{2} k_{tcij} \sum_{r=1}^{n-1} \sum_{q=1}^{m-1} \mathrm{R}^i_{r,q} \mathrm{R}^j_{n-r,m-q} - \sum_{i=1}^{2} \mathrm{B}_i \qquad \text{(S5)}$$

$$\mathrm{A}_i = \sum_{j=1}^{2} k_{fmij} \mathrm{M}_j + (k_{tcij} + k_{tdij}) \mathrm{R}^j_{0,0} + \sum_{j=1}^{2} k_{fpij} \sum_{r=0}^{\infty} \sum_{q=0}^{\infty} r^{i\delta(i-1)} q^{i-1} \mathrm{D}_{r,q}; i = 1,2 \qquad \text{(S6)}$$

$$\mathrm{B}_i = \sum_{j=1}^{2} k_{fpij} \mathrm{R}^j_{0,0} n^{i\delta(i-1)} m^{i-1} \mathrm{D}_{n,m}; i = 1,2 \qquad \text{(S7)}$$

where : $\mathrm{R}^i_{0,0}$ is the total concentration of the i-th type radicals: $\mathrm{R}^i_{0,0} = \sum_{r=0}^{\infty} \sum_{q=0}^{\infty} \mathrm{R}^i_{r,q}$

and $\delta(n,m) = \delta(n)\, \delta(m)$ is the Kronecker delta

2. Rate Functions for the Moment Equations of the Joint Chain Length-Copolymer Composition distribution

$$\mathrm{r}_{\lambda^i_{nm}} = \left(k_{iI} \mathrm{R}^\bullet \mathrm{M}_i + \sum_{j=1}^{2} k_{fmji} \mathrm{M}_i \lambda^i_{0,0} \right) \delta(m) + \sum_{j=1}^{2} k_{pji} \mathrm{M}_i \left[(2-i) \sum_{r}^{n} \binom{n}{r} \lambda^j_{rm} + (i-1) \sum_{r}^{m} \binom{r}{m} \lambda^j_{nr} \right] - \sum_{j=1}^{2} k_{pji} \mathrm{M}_j \lambda^i_{nm} - \mathrm{A}'_i \lambda^i_{nm} + \mathrm{B}_i + 3k_{th} \mathrm{M}_2^3 \delta(n+i-2, m+1-i) \quad ; \; n,m = 0,1 \qquad \text{(S8)}$$

$$\mathrm{r}_{\mu_{nm}} = \sum_{j=1}^{2} k_{pji} \mathrm{M}_i \left[(2-i) \sum_{r}^{n} \binom{n}{r} \lambda^j_{rm} + (i-1) \sum_{r}^{m} \binom{r}{m} \lambda^j_{nr} \right] - \sum_{j=1}^{2} k_{pji} \mathrm{M}_j \lambda^i_{nm} - \sum_{j=1}^{2} \sum_{i=1}^{2} k_{tcij} \lambda^j_{00} \lambda^i_{nm} + \frac{1}{2} \sum_{i=1}^{2} \sum_{j=1}^{2} k_{tcij} \sum_{r=1}^{n} \sum_{q=1}^{m} \binom{n}{r} \binom{m}{q} \lambda^i_{rq} \lambda^j_{n-r,m-q} \qquad \text{(S9)}$$

$$\mathrm{A}'_i = \sum_{j=1}^{2} \left(k_{fmij} \mathrm{M}_j + (k_{tcij} + k_{tdij}) \lambda^j_{00} \right) + \sum_{j=1}^{2} k_{fpij} \sum_{r=0}^{\infty} \sum_{q=0}^{\infty} \mu_{10}{}^{i\delta(i-1)} \mu_{01}{}^{i-1}; i = 1,2 \qquad \text{(S10)}$$

$$\mathrm{B}_i = \sum_{j=1}^{2} k_{fpij} \lambda_{00}^{j} \mu_{n+1,m}^{i\delta(i-1)} \mu_{n,m+1}^{(i-1)} ; i = 1,2 \quad \text{(S11)}$$

3. Variation of the reaction volume

The volume of the reacting mixture (*V*) was calculated by the following equation:

$$\frac{1}{\mathrm{V}} \frac{d\mathrm{V}}{d\mathrm{t}} = \frac{-\varepsilon (dX_{cum} / d\mathrm{t})}{1 - \varepsilon \mathrm{X}_{cum}} \quad \text{(S12)}$$

$$\text{with} \qquad \varepsilon = \frac{\rho_p - \rho_m}{\rho_p} \qquad \mathrm{X}_{cum} = \sum_{i=1}^{2} \mathrm{M}_i \mathrm{VMW}_i \Big/ \sum_{i=1}^{2} \mathrm{M}_{i0} \mathrm{V}_0 \mathrm{MW}_i \quad \text{(S13)}$$

Where ρ_p is the density of co-polymer, directly calculated from the homopolymers densities and the mean copolymer composition and ρ_m is the monomer's mixture density calculated from the monomer's densities by using a simple addition rule.

© 2019 by the authors. Submitted for possible open access publication under the terms and conditions of the Creative Commons Attribution (CC BY) license (http://creativecommons.org/licenses/by/4.0/).
